# Pre-pregnancy body mass index, gestational diabetes mellitus, and gestational weight gain: individual and combined effects on fetal growth

**DOI:** 10.3389/fpubh.2024.1354355

**Published:** 2024-03-11

**Authors:** Yanyu Lyu, Mingming Cui, Lingling Zhang, Guang Zheng, Hanxiao Zuo, Qingyong Xiu, Prakesh S. Shah

**Affiliations:** ^1^Experiment Center, Capital Institute of Pediatrics, Beijing, China; ^2^Beijing Municipal Key Laboratory of Child Development and Nutriomics, Capital Institute of Pediatrics, Beijing, China; ^3^Robert and Donna Manning College of Nursing and Health Sciences, University of Massachusetts Boston, Boston, MA, United States; ^4^School of Information Science and Engineering, Lanzhou University, Lanzhou, China; ^5^School of Public Health, University of Alberta, Edmonton, AB, Canada; ^6^Department of Pediatrics, Beijing Daxing Maternal and Child Care Hospital, Beijing, China; ^7^Department of Pediatrics, Mount Sinai Hospital, Toronto, ON, Canada

**Keywords:** pre-pregnancy body mass index, gestational diabetes mellitus, gestational weight gain, large-for-gestational-age, preterm birth, birth cohort, mediation analysis

## Abstract

**Background:**

Pre-pregnancy body mass index (BMI), gestational diabetes mellitus (GDM), and gestational weight gain (GWG) are interlinked and may play a complex role in fetal growth. We aimed to examine the relationship between pre-pregnancy BMI, GDM, GWG, and fetal growth outcomes and explore the contribution of GDM and GWG to the relationship between Pre-pregnancy obesity/overweight and large-for-gestational-age (LGA) in a prospective cohort.

**Methods:**

We prospectively recruited women in the first trimester and having one-step GDM screened with a 75-g oral glucose tolerance test between 24 and 28 weeks of gestation (*n* = 802). Outcomes included LGA, small-for-gestational-age (SGA), and preterm birth. To assess the individual and cumulative associations between pre-pregnancy BMI, GDM, GWG, and these outcomes, we used multivariate logistic regression analysis. Furthermore, we employed structural equation modeling (SEM) to investigate the mediating role of GDM and excessive GWG in the correlation between pre-pregnancy overweight/obesity and LGA.

**Results:**

Pre-pregnancy obesity, GDM, and excessive GWG were all independently associated with increased odds of LGA. Inadequate GWG was associated with higher odds of preterm birth. Compared with women unexposed to pre-pregnancy overweight/obesity, GDM, or excessive GWG, women exposed any two conditions had higher odds for LGA (AOR 3.18, 95% CI 1.25–8.11) and women with coexistence of all had the highest odds for LGA (AOR 8.09, 95% CI 2.18–29.97). The mediation analysis showed that GDM explained 18.60% (*p* < 0.05) of the total effect of pre-pregnancy overweight/obesity on LGA, and GWG explained 17.44% (*p* < 0.05) of the total effect.

**Conclusion:**

Pre-pregnancy obesity/overweight, GDM, and excessive GWG are associated with higher odds of fetal growth disturbances as individual factors and when they co-exist. The effect of pre-pregnancy overweight/obesity on LGA is partially achieved through GDM and excessive GWG.

## 1 Introduction

The prevalence of obesity and diabetes is increasing worldwide. The China Chronic Disease and Risk Factors Surveillance reported that the prevalence of obesity increased from 14.1% in 2013 to 16.5% in 2018, and the estimated prevalence of diabetes increased from 10.9% in 2013 to 12.4% in 2018 among adults (1). A population-based study in Tianjin reported that the prevalence of pre-pregnancy overweight and obesity was 19.5 and 6.3%, respectively, between 2010 and 2012 (2). Pre-pregnancy overweight/obesity is associated with an increased risk of adverse maternal and neonatal outcomes, such as preeclampsia, cesarean section, shoulder dystocia, preterm birth, large-for-gestational-age (LGA), and macrosomia (3–5). A systematic review reported a positive correlation between gestational diabetes mellitus (GDM) and adverse fetal growth outcomes such as macrosomia, intrauterine growth retardation (IUGR), and low birth weight (LBW) in south Asia (6). Gestational weight gain (GWG) is a complex fetal-maternal physiological phenomenon linked with women’s metabolic and nutritional status. Alterations in GWG are associated with fetal growth, preterm birth, cesarean section, GDM, and hypertensive disorders in pregnancy (7).

Pre-pregnancy body mass index (BMI), GDM, and GWG are correlated and can have independent and cumulative effects on fetal growth and pregnancy outcomes. It was reported that for every 1 kg/m^2^ increase in pre-pregnancy BMI, the prevalence of GDM is shown to increase by 0.92% (8). Approximately 50% of pregnant women exceeded their optimal weight gain, with women who have overweight or obesity having the highest prevalence of excessive GWG (9). Some studies have revealed an association between one or two of pre-pregnancy BMI, GDM, and GWG and birth outcomes (10–12). However, few data describe the magnitude of the association of all these risk factors alone or in different combinations with adverse birth outcomes. Bianchi et al. reported that GWG was associated with increased risk of macrosomia and LGA and decreased risk of preterm birth, whereas pre-pregnancy BMI and GDM were not independent risk factors for these adverse outcomes (13). Kim et al. reported that GDM contributed the least to LGA for all race or ethnic groups, whereas excessive GWG contributed the most (14). However, another study reported that fetal overgrowth was caused more by pre-pregnancy overweight and obesity than by GDM (15). A retrospective population-based study in China showed that pre-pregnancy obesity, GDM, and excessive GWG were all associated with higher odds of LGA and macrosomia; however, cumulative associations were not studied (16). Most of these studies have been retrospective designs and the prevalence of pre-pregnancy overweight/obesity or GDM are often underestimated and the rate of excessive GWG is overestimated, which would result in an inaccurate estimation of relative risk. In addition, there has been a lack of standard of recommendation for optimal GWG in China. Previous studies from China have been used the GWG recommendations of the Institute of Medicine (IOM). But American standards do not apply to Chinese women who are smaller than their American counterparts.

We aimed to examine the relationship between pre-pregnancy BMI, GDM, GWG, and fetal growth outcomes in a prospective cohort. Pre-pregnancy BMI affects GDM and GWG, which impact birth outcomes. We conducted a mediation analysis to further understand this relationship and provide insights for future studies.

## 2 Materials and methods

### 2.1 Study population

This study was conducted within a prospective cohort, which initiated to investigate the association of maternal overweight/obesity with infant growth and neurocognitive development from 2016 in Beijing Daxing Maternal and Child Care Hospital (17). Nine hundred eighty-six participants were recruited in the first trimester and 810 followed to delivery. Between 24 and 28 weeks of gestation, pregnant women were required to have one-step GDM screened with a 75-g oral glucose tolerance test. Women who had a twin pregnancy or stillbirth, and had preexisting diabetes were excluded in this study. The current study conformed to the principles drafted in the Helsinki declaration and was approved by the Ethical Committee of Capital Institute of Pediatrics (ref. number: SHERLL-2016034), and written informed consent was obtained from each participant.

### 2.2 Exposures, outcomes, and covariables

All participants were screened for GDM using a 75-g oral glucose tolerance test (OGTT) between 24–28 weeks of gestation as part of routine care. Venous blood samples were collected at 0, 1, and 2 h after a 75-g glucose load. Glycated hemoglobin (HbA1c) was tested at 0 h (fasting). If one or more of the blood glucose levels met or exceeded the pre-defined levels (0 h (fasting) ≥ 5.10 mmol/L; 1 h ≥ 10.00 mmol/L; 2 h ≥ 8.50 mmol/L), then women were diagnosed with GDM according to the recommendations of the International Association of the Diabetes and Pregnancy Study Groups (IADPSG) Consensus Panel (18). One month after OGTT, a blood sample was obtained to measure the lipid profile of each participant. The pre-pregnancy BMI was calculated using self-reported pre-pregnancy weight and height measured using a standard stadiometer with the participants standing barefoot at enrollment. All anthropometric measurements were taken by trained research personnel following a standardized protocol to ensure accuracy and consistency. Based on World Health Organization (WHO)-Asian criteria, women were classified as underweight (BMI < 18.5 kg/m^2^), normal weight (18.5–23.9 kg/m^2^), overweight (24.0–27.9 kg/m^2^), and obesity (BMI ≥ 28.0 kg/m^2^) (19). GWG was calculated as the difference between body weight at birth and self-reported pre-pregnancy weight. Based on the recommendations of optimal GWG for Chinese women, the range of adequate GWG was 11–16 kg for women with a pre-pregnancy BMI of <18.5; 8–14 kg for women with a pre-pregnancy BMI of 18.5–23.9; and 7–11 kg for women with a pre-pregnancy BMI of 24–27.9; and 5–9 kg for women with a pre-pregnancy BMI of ≥28.0 (20).

The primary outcomes were LGA, small-for-gestational-age (SGA), and preterm gestation. According to the Fenton growth chart, SGA and LGA were defined as birth weight lower than the 10th percentile or higher than the 90th percentile for gestational age, respectively (21). Gestational age was determined as the best estimate according to the hierarchy of first trimester ultrasound, last menstrual period, and obstetric estimate. Preterm birth was defined as gestational age at delivery <37 weeks.

Maternal sociodemographic information, including age, education, ethnicity, nativity, medical payment, and reproductive history, including parity and history of cesarean section, was obtained using a standardized questionnaire survey at the enrollment or from the medical chart.

### 2.3 Statistical analysis

The study population was described descriptively. The Student’s *t*-test, ANOVA, or Wilcoxon test were used for continuous variables, and the chi-square or Fisher’s exact test was used for categorical variables. Logistic regression models were conducted to examine the independent association of exposures (pre-pregnancy BMI, GDM, and GWG) with outcomes and adjusted for potential confounders, including maternal age, education, parity, nativity, medical payment, history of cesarean section, and infant sex (for preterm birth). To examine the individual and combined effects of pre-pregnancy BMI, GDM, and GWG on outcomes, we categorized women into four mutually exclusive groups: (1) Non-GDM, BMI <24, and no excessive GWG (reference group); (2) any one of GDM, BMI ≥24, excessive GWG; (3) any combination of two of GDM, BMI ≥24, excessive GWG; (4) GDM, BMI ≥24, and excessive GWG. Multiple logistic regression models were used to calculate adjusted odds ratios (AORs) and corresponding 95% confidence intervals (CIs) for the association between mutually exclusive combinations of exposures and outcomes.

**Figure 1:**
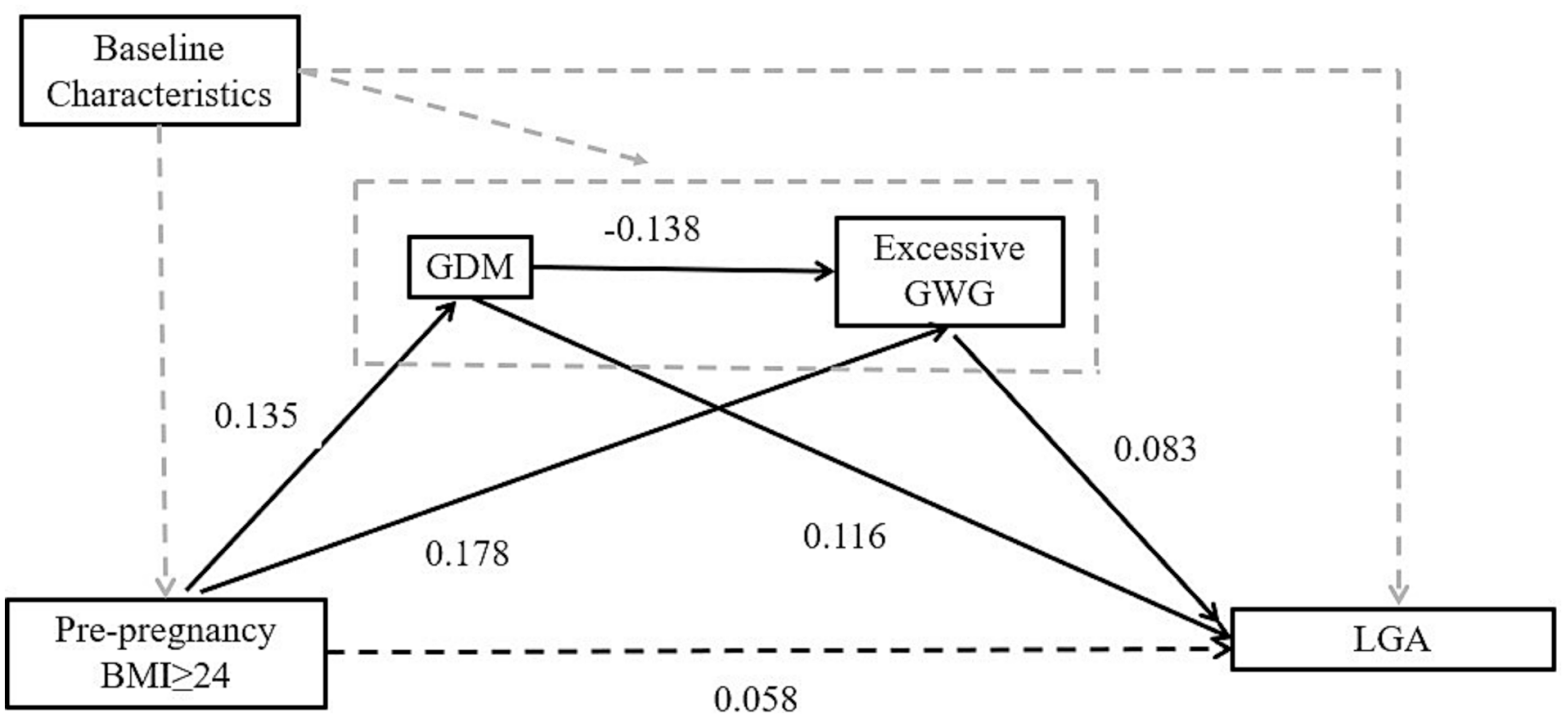
Schematic diagram showing the hypothesis that GDM and excessive GWG are two potential mediators in causal ways between pre-pregnancy BMI and outcome. Baseline Characteristics: age, education, parity, birth region, type of medical payment. The dashed black lines indicate paths that were estimated but were not statistically significant. GDM, gestational diabetes mellitus; BMI, body mass index; GWG, gestational weight gain; LGA, large for gestational age.

The possible mediations of GDM and excessive GWG between pre-pregnancy overweight/obesity and LGA were explored by using a structural equation model with the “lavaan” package in R statistical software (22). A bootstrap test was performed to test the simple mediating effect and the multiple chain mediated effect of GDM and excessive GWG with parameter “bootstrap = 1,000” (similar convergence results can also be obtained with other parameters, e.g., “bootstrap = 2000”). After simulation and parameter estimation, the regression coefficients, 95% CIs and *p* value were extracted from the model and reported in this study. Figure 1 presents the mechanistic pathways between pre-pregnancy overweight/obesity and LGA, indicating the indirect effects through GDM, excessive GWG, and the chain of GDM leading to excessive GWG.

All statistical analyses were performed using R statistical software version 3.5.1 (R Project for Statistical Computing).^1^ *p* < 0.05 was used to indicate statistically significant differences.

## 3 Results

A total of 986 pregnant women were approached and recruited before 13 weeks of gestation. During the follow-up phase, 51 women declined to continue, 94 left Beijing or went back to their hometown to give birth, and 31 had spontaneous abortions. The remaining 810 women were followed up until delivery. Of these, three women were identified later with preexisting diabetes, one delivered a stillbirth, and four with twins were excluded. Therefore, 802 pregnant women with live-born singletons were included in the final analysis. The mean maternal age in this study was 30.1 years, with a range spanning from 19 to 43 years. It is worth noting that 0.12% of the participants had chronic hypertension, 2.12% experienced gestational hypertension, 1.62% developed pre-eclampsia, and 4.49% suffered from anemia during pregnancy.

Maternal characteristics of included women and the incidences of fetal growth and pregnancy outcomes are reported in Table 1. A total of 14.1% of the included women were diagnosed with GDM. The mean [standard deviation (SD)] pre-pregnancy BMI was 22.6 (3.6) kg/m^2^, with overweight and obesity observed in 21.8 and 8.9%, respectively. The median GWG was 14.0 kg (Interquartile range (IQR): 11.5–17.0), with excessive GWG present in 57.2% of included women. Among infants, the mean (SD) gestational age was 39.4 (1.3) weeks, and the mean (SD) birthweight was 3370 (434) g. A total of 5.2% of infants were LGA, and 6.0% were SGA. The preterm birth rate was 2.2%.

**Table 1:** Maternal characteristics in GDM and pre-pregnancy BMI categories.

Groups	Total (*n* = 802)	Non-GDM (*n* = 689)	GDM (*n* = 113)	*p*	Underweight (*n* = 78)	Normal (*n* = 478)	Overweight (*n* = 175)	Obesity (*n* = 71)	*p*
**Maternal characteristic**									
Age (year)	30.1 ± 3.8	30.0 ± 3.7	30.8 ± 4.0	0.02	28.4 ± 3.5	30.3 ± 3.7	30.2 ± 3.8	30.6 ± 4.3	<0.01
<25	45 (5.6)	42 (6.1)	3 (2.7)	0.20	10 (12.8)	22 (4.6)	9 (5.1)	4 (5.6)	0.05
25–	332 (41.4)	290 (42.1)	42 (37.2)		44 (56.4)	193 (40.4)	66 (37.7)	29 (40.9)	
30–	323 (40.3)	274 (39.8)	49 (43.4)		17 (21.8)	196 (41.0)	83 (47.4)	27 (38.0)	
35–	102 (12.7)	83 (12.1)	19 (16.8)		7 (9.0)	67 (14.0)	17 (9.7)	11 (15.5)	
**Education**									
Middle-school or less	93 (11.7)	76 (11.2)	17 (15.2)	0.08	7 (9.0)	55 (11.7)	24 (13.7)	7 (9.9)	<0.01
High school	201 (25.4)	172 (25.3)	29 (25.9)		17 (21.8)	98 (20.9)	59 (33.7)	27 (38.0)	
College	236 (29.8)	196 (28.8)	40 (35.7)		25 (32.1)	129 (27.5)	55 (31.4)	27 (38.0)	
University graduate or more	263 (33.2)	237 (34.8)	26 (23.2)		29 (37.2)	187 (39.9)	37 (21.1)	10 (14.1)	
Ethnicity—Han	767 (95.6)	658 (95.5)	109 (96.5)	0.81	75 (96.2)	457 (95.6)	166 (94.9)	69 (97.2)	0.92
**Nativity**									
Beijing	274 (34.2)	236 (34.3)	38 (33.6)	0.96	21 (26.9)	132 (27.6)	81 (46.3)	40 (56.3)	<0.01
Hebei Province	204 (25.4)	174 (25.3)	30 (26.6)		17 (21.8)	121 (25.3)	44 (25.1)	22 (31.0)	
Other provinces	324 (40.4)	279 (40.5)	45 (39.8)		40 (51.3)	225 (47.1)	50 (28.6)	9 (12.7)	
**Medical payment**									
Reproductive health insurance	501 (62.5)	431 (62.6)	70 (62.0)	0.11	50 (64.1)	305 (63.8)	107 (61.1)	39 (54.9)	0.03
Out-of-pocket	215 (26.8)	190 (27.6)	25 (22.1)		20 (25.6)	133 (27.8)	43 (24.6)	19 (26.8)	
Others	86 (10.7)	68 (9.9)	18 (16.0)		8 (10.3)	40 (8.4)	25 (14.3)	13 (18.3)	
History of Cesarean section	166 (20.7)	139 (20.2)	27 (23.9)	0.37	6 (7.7)	84 (17.6)	53 (30.3)	23 (32.4)	<0.01
Pre-pregnancy BMI (mean ± SD, kg/m^2^)	22.6 ± 3.6	22.4 ± 3.5	24.1 ± 3.9	<0.01					
Underweight (BMI <18.5)	78 (9.7)	72 (10.5)	6 (5.3)	<0.01	–	–	–	–	
Normal (BMI 18.5–23.9)	478 (59.6)	423 (61.4)	55 (48.7)		–	–	–	–	
Overweight (BMI 24–27.9)	175 (21.8)	146 (21.2)	29 (25.7)		–	–	–	–	
Obese (BMI ≥28)	71 (8.9)	48 (7.0)	23 (20.4)		–	–	–	–	
Nulliparity	423 (52.7)	362 (52.5)	61 (54.0)	0.78	56 (71.8)	252 (52.7)	86 (49.1)	29 (40.9)	<0.01
GDM	–	–	–	–	6 (7.7)	55 (11.5)	29 (16.6)	23 (32.4)	<0.01
GWG in Kg [median (IQR)]	14.0 (11.5–17.0)	15.0 (12.0–17.5)	12.5 (10.0–15.0)	<0.01	15.0 (13.0–20.0)	15.0 (12.0–17.0)	13.0 (10.0–16.0)	12.0 (7.0–15.0)	<0.01
Inadequate	35 (4.4)	23 (3.3)	12 (10.6)	<0.01	7 (9.0)	9 (1.9)	11 (6.3)	8 (11.3)	<0.01
Adequate	308 (38.4)	256 (37.2)	52 (46.0)		36 (46.2)	215 (45.0)	42 (24.0)	15 (21.1)	
Excessive	459 (57.2)	410 (59.5)	49 (43.4)		35 (44.9)	254 (53.1)	122 (69.7)	48 (67.6)	
Preeclampsia^#^	13 (1.6)	11 (1.6)	2 (1.8)	0.70	0 (0.0)	6 (1.3)	6 (3.4)	1 (1.4)	0.13
Cesarean section	302 (37.7)	248 (36.0)	54 (47.8)	0.02	19 (24.4)	156 (32.6)	90 (51.4)	37 (52.1)	<0.01
Postpartum hemorrhage	258 (32.2)	219 (31.8)	39 (34.5)	0.57	19 (24.4)	150 (31.4)	58 (33.1)	31 (43.7)	0.08
**Outcomes**									
LGA	42 (5.2)	29 (4.2)	13 (11.5)	<0.01	4 (5.1)	18 (3.8)	10 (5.7)	10 (14.1)	<0.01
SGA	48 (6.0)	43 (6.2)	5 (4.4)	0.45	6 (7.7)	29 (6.1)	9 (5.1)	4 (5.6)	0.89
Preterm	18 (2.2)	12 (1.7)	6 (5.3)	0.03	1 (1.3)	9 (1.9)	6 (3.4)	2 (2.8)	0.61

Continuous variables are presented as mean ± standard deviation (SD) or medians (25th–75th percentile) as appropriate and categorical variables as *n* (%). GDM, gestational diabetes mellitus; BMI, body mass index; GWG, gestational weight gain; IQR, interquartile range; LGA, large for gestational age; SGA, small for gestational age.

Compared to the non-GDM group, the GDM group had significantly higher maternal age, pre-pregnancy BMI, the proportion of cesarean section, a higher rate of preterm and LGA infants, and lower GWG. Comparison of related characteristics of maternal pre-pregnancy BMI groups, the maternal age, education, nativity, medical payment, nulliparity, history of cesarean section, GDM, GWG, and LGA of the four groups was significantly different.

There were no significant differences in neonatal anthropometric indexes, including birthweight, body length, and head, scapular, and chest circumferences between GDM and non-GDM groups. In addition, the offspring of women classified as having obesity based on pre-pregnancy BMI had higher birthweight than that of women classified as normal. Compared with neonates of mothers with adequate GWG, the averages of five anthropometric indexes at birth were all higher in neonates of mothers with excessive GWG, whereas these anthropometric indexes were all lower in the neonates of mothers with inadequate GWG (all *p* < 0.05). Neonatal anthropometric measurements based on categories of maternal pre-pregnancy BMI, GDM, and GWG are shown in Supplementary Table S1.

Metabolic features of women using GDM and pre-pregnancy BMI classification groups are reported in Supplementary Table S2. In the second trimester, women with GDM had significantly higher levels of fasting glucose, 1 h-OGTT glucose, 2 h-OGTT glucose, and HbA1c (all *p* < 0.0001). Early in the third trimester, they had higher levels of triglycerides (*p* = 0.0104) and remnant cholesterol (*p* = 0.0235) and lower level of high-density lipoprotein (HDL) cholesterol (*p* < 0.0001) and total cholesterol (*p* = 0.0471) compared to those without GDM. The levels of these metabolic indices, except remnant cholesterol, were significantly different across the four pre-pregnancy BMI groups. In comparison to women with pre-pregnancy normal weight, those with pre-pregnancy overweight or obesity had significantly higher levels of fasting glucose, 1 h-OGTT glucose, 2 h-OGTT glucose, HbA1c, and triglycerides and lower levels of total cholesterol, HDL cholesterol, and low-density lipoprotein (LDL) cholesterol (all *p* < 0.05).

Odds ratio (OR) and adjusted odds ratio (AOR) for fetal growth outcomes associated with GDM, pre-pregnancy BMI, and inadequate/excessive GWG are reported in Table 2. GDM was independently associated with higher odds of LGA [AOR 2.67; 95% confidence interval (CI) 1.27–5.64]. Compared with infants born to mothers with pre-pregnancy normal weight, those born to mothers with obesity had higher odds of LGA. Women with excessive GWG had higher odds of delivering LGA. Mothers with inadequate GWG had higher odds of preterm birth.

**Table 2:** Association between GDM, pre-pregnancy BMI, and GWG and outcomes.

Exposure	LGA (*n* = 42)		SGA (*n* = 48)		Preterm birth (*n* = 18)	
	OR (95% CI)	AOR (95% CI)	OR (95% CI)	AOR (95% CI)	OR (95% CI)	AOR (95% CI)
**GDM**						
No	1	1	1	1	1	1
Yes	2.96 (1.49–5.88)	2.67 (1.27–5.64)	0.70 (0.27–1.80)	0.61 (0.23–1.65)	3.16 (1.16–8.61)	2.13 (0.71–6.41)
**Pre-pregnancy BMI**						
Underweight	1.38 (0.46–4.20)	1.39 (0.44–4.36)	1.29 (0.52–3.22)	1.10 (0.43–2.83)	0.68 (0.09–5.42)	0.88 (0.11–7.38)
Normal	1	1	1	1	1	1
Overweight	1.55 (0.70–3.42)	1.43 (0.61–3.36)	0.84 (0.39–1.81)	0.71 (0.32–1.59)	1.85 (0.65–5.28)	1.46 (0.46–4.61)
Obesity	4.19 (1.85–9.49)	4.43 (1.69–11.59)	0.92 (0.32–2.71)	0.67 (0.20–2.22)	1.51 (0.32–7.14)	0.60 (0.10–3.63)
**GWG**						
Inadequate	2.01 (0.42–9.71)	1.20 (0.22–6.36)	1.16 (0.33–4.09)	1.15 (0.31–4.24)	10.75 (3.52–32.85)	8.80 (2.63–29.44)
Adequate	1	1	1	1	1	1
Excessive	2.41 (1.13–5.13)	2.47 (1.12–5.42)	0.62 (0.34–1.14)	0.56 (0.30–1.05)	0.38 (0.11–1.30)	0.37 (0.10–1.34)

Multivariable logistic regression models were adjusted for maternal age, education, parity, nativity, medical payment, history of cesarean section, and infant sex (for preterm birth). We also adjusted for the other exposures not measured in each model (i.e., if modeling GDM, we adjusted for pre-pregnancy BMI and GWG; if modeling pre-pregnancy BMI, we adjusted for GDM and GWG; if modeling GWG, we adjusted for pre-pregnancy BMI and GDM). GDM, gestational diabetes mellitus; BMI, body mass index; GWG, gestational weight gain; SGA, small for gestational age; LGA, large for gestational age; OR, odds ratio; AOR, adjusted odds ratio; CI, confidence interval.

The results of outcomes in different combinations of exposures compared between non-overweight/obesity, non-GDM, and no excessive GWG women are reported in Table 3. Compared with women unexposed to overweight/obesity, GDM, or excessive GWG, women exposed any two of these three risk factors had higher odds for LGA (AOR 3.18, 95% CI 1.25–8.11), and women with the coexistence of overweight/obesity, GDM, and excessive GWG had the highest odds for LGA (AOR 8.09, 95% CI 2.18–29.9). Furthermore, regarding different groups of combinations of exposures and LGA risk, a clear trend of increased risk was observed after adjustment (P_trend_ = 0.0004).

**Table 3:** Individual and combined association of pre-pregnancy overweight/ obesity, GDM, and excessive GWG with LGA.

Groups		LGA (*n* = 42)	
	No.	OR (95% CI)	AOR (95% CI)
Non-GDM, BMI < 24, no excessive GWG	233	1.00 (ref)	1.00 (ref)
any one of GDM, BMI >24, excessive GWG	348	1.05 (0.40–2.74)	1.11 (0.42–2.93)
any combination of two of GDM, BMI ≥24, excessive GWG	199	3.38 (1.39–8.21)	3.18 (1.25–8.11)
GDM, BMI ≥24, excessive GWG	24	8.42 (2.44–29.09)	8.09 (2.18–29.97)

The Multivariable logistic regression model was adjusted for maternal age, education, parity, nativity, medical payment, and history of cesarean section. GDM, gestational diabetes mellitus; BMI, body mass index; GWG, gestational weight gain; LGA, large for gestational age; OR, odds ratio; AOR, adjusted odds ratio; CI, confidence interval.

In the mediation analysis via the “lavaan” package, after adjusting for covariates, including maternal age, education, parity, birth region and type of medical payment, pre-pregnancy overweight/obesity (BMI ≥ 24) has a positive total effect on LGA. The total effect size is 0.086, and this effect is statistically significant (*p* = 0.022). The direct effect of pre-pregnancy overweight/obesity on LGA is 0.058, but it is not statistically significant (*p* = 0.122). The indirect effect of pre-pregnancy overweight/obesity on LGA is 0.029, and it is statistically significant (*p* = 0.003). This indirect effect is achieved through three pathways. The first pathway is through GDM. The single mediating effect size of GDM on LGA is 0.016 (*p* = 0.012). The second pathway is through excessive GWG. The single mediating effect size of excessive GWG on LGA is 0.015 (*p* = 0.015). The third pathway is through a chain of GDM leading to excessive GWG, and then to LGA. However, the results of the multiple chain mediated model showed a small negative chain mediating effect is −0.002 and not statistically significant by GDM and excessive GWG (*p* = 0.067). Approximately 33.72% of the impact of pre-pregnancy overweight/obesity on LGA is achieved indirectly through two mediation variables GDM and GWG (see Table 4 and Figure 1).

**Table 4:** Total, direct and indirect effects of pre-pregnancy overweight/obesity (BMI ≥ 24) on LGA, with mediation though GDM, excessive GWG, GDM and excessive GWG pathway.*

	β	SE	95% CI	*p*	Proportion mediated
Total effect	0.086	0.038	0.012 ~ 0.160	0.022	100%
Direct effect	0.058	0.037	−0.015 ~ 0.131	0.122	67.44%
Indirect effect	0.029	0.010	0.010 ~ 0.048	0.003	33.72%
BMI → GDM → LGA	0.016	0.008	0.000 ~ 0.031	0.048	18.60%
BMI → GWG → LGA	0.015	0.006	0.003 ~ 0.027	0.015	17.44%
BMI → GDM → GWG → LGA	−0.002	0.001	−0.003 ~ 0.000	0.067	−2.33%

GDM, gestational diabetes mellitus; BMI, body mass index; GWG, gestational weight gain; LGA, large for gestational age; CI, confidence interval, SE, standard error.

## 4 Discussion

This prospective study found that pre-pregnancy obesity, GDM, and excessive GWG are independently associated with LGA births. Women with two or more of these exposures have an increased risk of LGA compared to unexposed women. The analysis revealed that GDM and excessive GWG partially mediate the relationship between pre-pregnancy overweight/obesity and LGA, accounting for 33.72% of the effect.

A Florida population-based study found that pre-pregnancy overweight/obesity, GDM, and excessive GWG independently increase the risk of LGA, with excessive GWG accounting for the highest percentage (33.3–37.7%) of LGA cases (14). Black et al. found that pre-pregnancy overweight/obesity was a larger contributor (21.6%) to LGA than GDM. They also observed an increasing trend of LGA prevalence with increasing GWG across all combinations of GDM and pre-pregnancy BMI categories, indicating an additive effect of GWG, pre-pregnancy BMI, and GDM on LGA risk (15). A meta-analysis of European, North American, and Australian cohorts showed that maternal overweight/obesity accounted for 23.9% of pregnancy complications, and excessive GWG was responsible for 31.6% of LGA cases (23). An Italian study found that GWG increases the risk of macrosomia and LGA while reducing the risk of preterm birth. However, GDM and pre-pregnancy BMI were not independent risk factors for these outcomes (13). In comparison to previous studies, our study adds to the literature by considering the cumulative effect of multiple risk factors on LGA and exploring the mediating roles of GDM and excessive GWG in this relationship. Furthermore, the study contributes to filling knowledge gaps in this field by quantifying the direct and indirect effects of pre-pregnancy overweight/obesity on LGA through mediation analysis.

Compared with normal-weight mothers, pre-pregnancy obesity was associated with a higher birth weight of infants and independently increased the odds of LGA in our study, which is consistent with the results of a systematic review (24). Fleten et al. reported that pre-pregnancy BMI had a greater impact than exercise during pregnancy on birthweight, with each unit increase in pre-pregnancy BMI leading to a 20.3 g increase in birth weight (25). We found that GDM and pre-pregnancy overweight/obesity were associated with abnormal glucose in the late of second trimester and lipid profiles in the early of third trimester. Elevated fasting glucose, 1 h-OGTT glucose, 2 h-OGTT glucose, HbA1c, and triglycerides and lower HDL cholesterol were identified in these groups. Additionally, GDM women had higher remnant cholesterol levels. Lipids are essential for fetal development and placental function, with triglycerides and remnant cholesterol positively with embryo size (26), birth weight, and the risk of LGA even after adjustment for glucose concentrations (27, 28). This is in line with the fetal over-nutrition hypothesis, which suggests that maternal lipids are crucial for fetal overgrowth apart from maternal glucose concentrations (29). The “Developmental origins of health and disease” theory suggests that early-life exposures, including preconception, pregnancy, and early postnatal periods, can increase the risk of diseases such as obesity, type 2 diabetes, insulin resistance, cardiovascular diseases, etc., later in life (30). Therefore, lifestyle intervention focusing on a healthy diet and physical exercise before pregnancy might promote a normal BMI and support the offspring’s long-term health.

The observed lower LDL cholesterol levels in the third trimester among pregnant women with pre-pregnancy overweight and obesity could be attributed to several factors. Pregnant women with pre-pregnancy overweight and obesity received weight management interventions initiated by outpatient obstetricians from early pregnancy to late pregnancy. These interventions included dietary management and appropriate physical activity aimed at rigorously controlling weight gain throughout pregnancy. The pregnant women may adopt dietary patterns that are lower in saturated fats and cholesterol, which could impact LDL cholesterol levels. Ramírez-Vélez et al. highlighted the impact of exercise during pregnancy on maternal lipid profiles, suggesting that physical activity interventions could influence lipid metabolism (31). Moreover, Chen et al. explored the relationship between physical activity and plasma lipid metabolism during pregnancy, emphasizing the role of objectively measured physical activity and sitting time (32). It is plausible that the weight management interventions may have influenced physical activity levels and, consequently, lipid metabolism, contributing to the observed lower LDL cholesterol levels in the third trimester. Further investigation is warranted to elucidate the specific mechanisms underlying this observation and its implications for maternal and fetal health in the context of the DOHaD framework. Our study showed that GDM was an independent risk factor for LGA, similar to a retrospective study in Xiamen, China (16). They also reported that GDM was associated with preterm birth. In our study, women with GDM had increased odds of preterm birth in unadjusted models; however, these associations disappeared after adjustment. Another prospective cohort study in China demonstrated that GDM was not a significant risk factor for all the studied adverse pregnancy outcomes, including preterm birth, LGA, and macrosomia, even after adjusting for pre-pregnancy BMI and GWG (33). The authors suggested that it might be due to the early screening and management of GDM in the study population and the difference in sample size and ethnicity. Currently, the most acceptable diagnostic criterion for GDM used in China is a 2-h 75 g OGTT performed during 24–28 gestational weeks according to the IADPSG diagnostic criteria. Once a pregnant woman is screened for GDM, a comprehensive intervention, including education on the basic knowledge of GDM, dietary intervention, physical exercise, weight management, and blood glucose self-monitoring methods, will be implemented for her by professional physicians and nurses (34). In our study, the GWG of GDM pregnant women was significantly lower than that of non-GDM women and GDM significantly negatively predicted excessive GWG (β = −0.138, *p* < 0.001) in the mediation analysis, which may reflect the possible intervention effect.

Our research found that women with insufficient GWG had greater odds of preterm birth, consistent with a previous report using similar criteria for GDM, pre-pregnancy BMI, and GWG classifications. In a study of 3,253 pregnant women in China, Teshome et al. reported that excessive GWG lowered the risk of preterm birth and SGA, and GWG was positively associated with fetal growth measurements before birth and neonatal measurements, including birthweight and length (33). However, they did not report the combined effects of pre-pregnancy overweight/obesity, GDM, and GWG on fetal growth outcomes. Another retrospective study in Beijing reported that pre-pregnancy normal weight women with inadequate GWG had an increased risk of LBW and preterm birth (35). A systematic review and meta-analysis reported that women with high GWG have lower unadjusted risks of preterm birth and LBW; however, high weekly GWG was associated with increased preterm birth (36). Due to these inconsistent results, more prospective analyses like our study are needed.

The study also conducted a mediation analysis to determine the impact of pre-pregnancy overweight/obesity (BMI ≥ 24) on LGA and identified a significant indirect effect through two mediators: GDM and excessive GWG. The indirect effect was achieved through two distinct pathways: one directly through GDM and the other through excessive GWG. The chain mediating effect from GDM to excessive GWG to LGA was not significant, suggesting that these factors operate independently rather than as a sequential pathway.

There are several strengths in our study. First, this is a prospective study with longitudinal follow-up of a medium group of pregnant women. We can collect additional relevant information that are not readily available in registers or health administration database and ensure more accuracy to the data. Second, we utilized the WHO-Asian criteria for BMI classification and the current recommended optimal GWG for Chinese women, adjusting for Asian-specific BMI categories, to accurately depict the maternal nutritional status before and during pregnancy. Third, we aimed to address the separately and combined effects of pre-pregnancy BMI, GDM, and GWG on fetal overgrowth and found that co-existing overweight/obesity, GDM, and excessive GWG significantly increased the risk of LGA. A causal mediation analysis revealed that GDM and excessive GWG partially mediate the relationship between pre-pregnancy overweight/obesity and LGA. However, this study also has limitations. First, pre-pregnancy weight was self-reported, which may lead to under-reporting (37). Second, the subgroup of co-existing exposures had few participants, leading to wide confidence intervals of odds ratio (38). Third, a limitation of our regression model is that we did not collect detailed information on dietary intake, and as a result, energy intake was not included as a significant confounding factor. Fourth, Beijing is a diverse and populous city in China. Therefore, our findings should be interpreted with caution, considering the specific population studied. Additionally, the decision to combine pre-pregnancy overweight and obesity categories in subsequent analyses may have masked potential distinctions between the two groups, thereby limiting our ability to discern nuanced associations. These limitations should be taken into consideration when interpreting the results of our study and underscore the need for further research in diverse populations to validate our findings and explore the distinct impacts of overweight and obesity on fetal outcomes.

## 5 Conclusion

Pre-pregnancy BMI, GDM, and GWG were all associated with higher odds of fetal growth disturbances. Pregnancy overweight/obesity, GDM and excessive GWG had cumulative effects of development of LGA. GDM and excessive GWG can indirectly affect the impact of pre-pregnancy overweight/obesity on LGA. Findings need to be verified with a larger sample size.

## Data availability statement

The original contributions presented in the study are included in the article/Supplementary material, further inquiries can be directed to the corresponding author.

## Ethics statement

The studies involving humans were approved by the Ethical Committee of Capital Institute of Pediatrics (ref. number: SHERLL-2016034). The studies were conducted in accordance with the local legislation and institutional requirements. The participants provided their written informed consent to participate in this study.

## Author contributions

YL: Methodology, Supervision, Writing – original draft, Conceptualization, Formal analysis, Funding acquisition, Writing – review & editing. MC: Data curation, Writing – review & editing. LZ: Methodology, Writing – review & editing. GZ: Formal analysis, Writing – review & editing. HZ: Investigation, Writing – review & editing. QX: Investigation, Writing – review & editing. PS: Writing – review & editing.

## Footnotes

^1^http://www.r-project.org
