# Supplementary material for: Pre-pregnancy body mass index, gestational diabetes mellitus, and gestational weight gain: individual and combined effects on fetal growth
Source: Front Public Health. 2024 Mar 11;12:1354355. doi: 10.3389/fpubh.2024.1354355 (PMC10961333; doi:10.3389/fpubh.2024.1354355)
Supplement: Supplementary file 1 [file Table_1.DOCX]

Supplementary Material

**Supplementary Table S1.** Growth measurements at birth in maternal exposure groups

| **Anthropometry** | **GDM** | |  | **Pre-pregnancy BMI** | | | |  | **GWG** | | |
| --- | --- | --- | --- | --- | --- | --- | --- | --- | --- | --- | --- |
|  | No  (n=689) | Yes  (n=113) |  | Underweight  (n=78) | Normal  (n=478) | Overweight  (n=175) | Obesity  (n=71) |  | Inadequate  (n=35) | Adequate  (n=308) | Excessive  (n=459) |
| Birth weight (g) | 3367±415 | 3388±535 |  | 3370±401 | 3346±422 | 3398±431 | 3462±537^a^ |  | 3071±682 ^b^ | 3299±412 | 3440±407 ^b^ |
| Birth length (cm) | 50.5±1.7 | 50.4±1.8 |  | 50.6±1.7 | 50.4 ±1.7 | 50.6±1.6 | 50.8±2.0 |  | 49.3±3.0 ^b^ | 50.3±1.8 | 50.8±1.4 ^b^ |
| Head circumference (cm) | 33.6±2.4 | 33.4±2.9 |  | 33.3±2.8 | 33.5 ±2.3 | 33.7±2.6 | 34.0±2.8 |  | 32.1±3.6 ^b^ | 33.2±2.4 | 34.0±2.3 ^b^ |
| Shoulder circumference (cm) | 35.1±2.6 | 35.1±3.0 |  | 34.8±2.7 | 35.0±2.5 | 35.2±2.8 | 35.6±3.0 |  | 33.5±3.6 ^b^ | 34.7±2.6 | 35.4±2.5 ^b^ |
| Chest circumference (cm) | 33.6±2.6 | 33.6±3.0 |  | 33.3±2.6 | 33.5±2.5 | 33.8±2.7 | 34.0±3.3 |  | 31.9±4.0 ^b^ | 33.2±2.6 | 33.9±2.5 ^b^ |

GDM, gestational diabetes mellitus; BMI, body mass index; GWG, gestational weight gain. The superscript a stands for statistically significant at P < 0.05 compared with the normal weight group using one-way ANOVA. The superscript b stands for statistically significant at P < 0.05 compared with adequate GWG group using one-way ANOVA.

**Supplementary Table 2. Metabolic features of women in GDM and pre-pregnancy BMI categories**

|  | Non-GDM  (n=689) | GDM  (n=113) | P |  | Underweight  (n=78) | Normal  (n=478) | Overweight  (n=175) | Obesity  (n=71) | P |
| --- | --- | --- | --- | --- | --- | --- | --- | --- | --- |
| **OGTT** |  |  |  |  |  |  |  |  |  |
| Fasting glucose (mmol/L) | 4.4±0.3 | 5.2±0.5 | **<0.01** |  | 4.4±0.3 | 4.5±0.4 | 4.7±0.4^a^ | 4.9±0.5^a^ | **<0.01** |
| 1h-OGTT glucose (mmol/L) | 6.8±1.3 | 9.1±1.7 | **<0.01** |  | 6.8±1.7 | 6.9±1.5 | 7.3±1.5 ^a^ | 8.1±1.8 ^a^ | **<0.01** |
| 2h-OGTT glucose (mmol/L) | 6.2±1.0 | 7.8±1.4 | **<0.01** |  | 6.0±1.1^a^ | 6.3±1.1 | 6.5±1.1 | 6.9±1.3 ^a^ | **<0.01** |
| HbA1c (%) | 5.3±0.4 | 5.5±0.4 | **<0.01** |  | 5.3±0.4 | 5.3±0.4 | 5.4±0.4 ^a^ | 5.4±0.4 | **<0.01** |
| **Lipid profiles** |  |  |  |  |  |  |  |  |  |
| Triglycerides (mmol/L) | 2.7±0.8 | 3.1±1.6 | **0.01** |  | 2.6±0.8 | 2.7±1 | 2.9±1.0 | 3.0±0.9 ^a^ | **0.01** |
| Total cholesterol (mmol/L) | 6.2±1.1 | 6.0±1.0 | **0.05** |  | 6.6±1.1 | 6.3±1 | 6.0±1.1 ^a^ | 5.7±0.8 ^a^ | **<0.01** |
| HDL cholesterol (mmol/L) | 2.0±0.4 | 1.8±0.4 | **<0.01** |  | 2.1±0.3 | 2.1±0.4 | 1.9±0.4 ^a^ | 1.8±0.3 ^a^ | **<0.01** |
| LDL cholesterol (mmol/L) | 3.3±0.8 | 3.1±0.8 | 0.08 |  | 3.6±0.9 ^a^ | 3.3±0.8 | 3.1±0.9 | 2.9±0.7 ^a^ | **<0.01** |
| Remnant cholesterol (mmol/L) | 0.93±0.29 | 1.04±0.47 | **0.02** |  | 0.95±0.31 | 0.95±0.33 | 0.93±0.34 | 0.95±0.25 | **0.89** |

The superscript a stands for statistically significant at P < 0.05 compared with the normal weight group using one-way ANOVA.Missing data for fasting glucose, 19; missing data for 1h-OGTT glucose, 28; missing data for 2h-OGTT glucose, 28; missing data for HbA1c, 25; missing data for lipid profiles, 52. GDM, gestational diabetes mellitus; BMI, body mass index; OGTT, oral glucose tolerance test; HbA1c, Glycated hemoglobin; HDL, high-density lipoprotein; LDL, low-density lipoprotein.
